# Context, mechanisms and outcomes in end of life care for people with advanced dementia

**DOI:** 10.1186/s12904-016-0103-x

**Published:** 2016-03-10

**Authors:** Nuriye Kupeli, Gerard Leavey, Kirsten Moore, Jane Harrington, Kathryn Lord, Michael King, Irwin Nazareth, Elizabeth L. Sampson, Louise Jones

**Affiliations:** Marie Curie Palliative Care Research Department, Division of Psychiatry, University College London, 6th Floor, Maple House, 149 Tottenham Court Road, London, W1T 7NF UK; Bamford Centre for Mental Health & Wellbeing, University of Ulster, Londonderry, UK; Division of Psychiatry, University College London, Londonderry, UK; Department of Primary Care & Population Health, University College London, Londonderry, UK; Barnet Enfield and Haringey Mental Health Trust Liaison Psychiatry Team, St. Anns Hospital, London, UK

**Keywords:** Dementia, Palliative care, Qualitative research, Realist framework, Care homes

## Abstract

**Background:**

The majority of people with dementia in the UK die in care homes. The quality of end of life care in these environments is often suboptimal. The aim of the present study was to explore the context, mechanisms and outcomes for providing good palliative care to people with advanced dementia residing in UK care homes from the perspective of health and social care providers.

**Method:**

The design of the study was qualitative which involved purposive sampling of health care professionals to undertake interactive interviews within a realist framework. Interviews were completed between September 2012 and October 2013 and were thematically analysed and then conceptualised according to context, mechanisms and outcomes. The settings were private care homes and services provided by the National Health Service including memory clinics, mental health and commissioning services in London, United Kingdom. The participants included 14 health and social care professionals including health care assistants, care home managers, commissioners for older adults’ services and nursing staff.

**Results:**

Good palliative care for people with advanced dementia is underpinned by the prioritisation of psychosocial and spiritual care, developing relationships with family carers, addressing physical needs including symptom management and continuous, integrated care provided by a multidisciplinary team. Contextual factors that detract from good end of life care included: an emphasis on financial efficiency over person-centred care; a complex health and social care system, societal and family attitudes towards staff; staff training and experience, governance and bureaucratisation; complexity of dementia; advance care planning and staff characteristics. Mechanisms that influence the quality of end of life care include: level of health care professionals’ confidence, family uncertainty about end of life care, resources for improving end of life care and supporting families, and uncertainty about whether dementia specific palliative care is required.

**Conclusions:**

Contextual factors regarding the care home environment may be obdurate and tend to negatively impact on the quality of end of life dementia care. Local level mechanisms may be more amenable to improvement. However, systemic changes to the care home environment are necessary to promote consistent, equitable and sustainable high quality end of life dementia care across the UK care home sector.

## Background

An increasing number of people in the UK with advanced dementia die in long term care settings [1], a trend noted across Belgium, the United States and Canada [2]. The advanced stage of dementia is characterised by double incontinence, inability to communicate needs and multiple co-morbidities such as diabetes and hypertension [3]. People with advanced dementia experience cognitive and physical decline often with complex symptoms such as pain and behavioural disturbances [4–7]. Compared to those who are more cognitively able, they are less likely to receive pain relief [8] and are more likely to be hospitalised due to infections [9]. Due to increasing severity of symptoms and failure of home care services to meet the needs of people with advanced dementia and their family carers [10], institutionalisation following a hospital admission is common [11]. However, care homes are not always equipped to provide proactive and coordinated ‘palliative’ care to those who are nearing the end of life [12–15].

People with dementia who are approaching death are less likely to have advance directives against unnecessary and burdensome interventions such as tube feeding compared to those dying with terminal cancer [16]. Inappropriate prescribing of medication at end of life is also common with 84.6 % of patients with advanced dementia prescribed more than one medication [17]. Evidence from our wider Compassion programme on end of life in advanced dementia [18] (National Institute for Health Research (NIHR) portfolio reference numbers: 12,621; 12,623) has shown that palliative care provision for those living in care homes requires a complex intervention consisting of multidisciplinary care using a continuous and integrated approach [15, 19] (Sampson et al: Living and dying with advanced dementia: A prospective cohort study, In preparation). This is supported by an emphasis on educating care home staff and other health care professionals [15, 20], in particular on the awareness of the dying phase which is difficult to recognise in dementia [21]. Although prognostication in dementia is difficult, tools such as the Advanced Dementia Prognostic Tool (ADEPT) [22] have been developed to predict survival of those residing in nursing homes and have been shown to have some promising results [23].

Long-term residential care for people with advanced dementia in the United Kingdom is usually provided in care homes which offer nursing care by qualified nurses supported by health care assistants who may have a vocational certificate. Seventy percent of care home residents have some form of dementia [24]. General Practitioners (GPs) commonly provide primary care to people with advanced dementia residing in care homes and residents have access to a variety of services offered by the National Health Service (NHS) including psychiatry, physiotherapy, speech and language therapy and occupational therapy. Funding for health services in the UK is managed at a local level via Clinical Commissioning Groups (CCGs). Some care homes are supported by specialist palliative care services based within the health care system whilst others must rely on the charitable sector such as outreach teams from hospices. Similarly, Admiral Nurses are specialist nurses for people with dementia and their families throughout the disease progression and into bereavement for families. This service is provided by a charity and therefore access across the UK is variable [25].

The health and social care system in the UK as outlined above may be seen as hindering the provision of continuous, integrated end of life care for people with advanced dementia. Integrated care for improving end of life care is predicated on good relationships between health care professionals (HCPs), multidisciplinary care by a team of experts, continuous care and streamlined referral processes with external services. The importance of, and barriers to, integrated palliative care have been described in our previous paper [15]. Our definition of what constitutes integrated palliative care also fits in with the definition of palliative care for people with advanced dementia as described in a key paper by the European Association of Palliative Care (EAPC) [26]. Palliative dementia care has been identified as continuous, proactive person-centred care with timely recognition of the dying phase whilst providing comfort, psychosocial and spiritual support and avoiding unnecessary burdensome treatments. The EAPC also emphasise the importance of the collaboration between HCPs and family carers when making end of life care decisions and the education of HCPs [26].

In this paper, we report on findings of qualitative interviews with commissioners and HCPs working in care homes. The interviews were conducted as part of a wider programme of work in which we used a realist approach [27] to inform the development and preliminary implementation of an intervention to improve care towards the end of life in advanced dementia [18, 19, 28]. Consistent with a realist approach, we began with a preliminary programme theory informed by expert opinion within the research team led by clinical academic specialists in dementia care and palliative care. Our thinking developed further through a rapid review of the literature. Our original programme theory of relevance for the work reported in this paper was: ‘That end of life care for people with end stage dementia could be improved by increasing the depth of understanding of symptoms and unmet health and social care needs, allowing prompt recognition and appropriate confident management by professionals, working in consultation with carers. This understanding should be underpinned by improved awareness amongst all groups of the natural history of dementia and the significance of the end stage’. We used this theory to inform the topics probed in our interactive qualitative interviews, the data arising from which are considered in this paper. Our analysis explores the context, mechanisms and outcomes that may explain our theory.

### Aims

To explore the context, mechanisms and outcomes for providing good palliative care to people with advanced dementia residing in UK care homes. Using the realist evaluation framework, we aimed to identify the range of contextual factors found within the care home and health care environments, how they influence various mechanisms affecting care in a positive or negative way, and how these mechanisms may either improve or undermine the outcomes related to end of life care provided to people with advanced dementia. This was done by broadly exploring health and social care providers’ recognition of dementia as a terminal illness, the management of acute medical problems, appropriateness of hospitalisation, the role of advance care plans and do not attempt resuscitation (DNAR) orders, and HCPs’ views of whether the health and social care system is meeting the needs of people with advanced dementia and their families.

## Methods

### Ethical review

Ethical approval was granted by the University College London Ethics Committee (Reference: 3578/001) on 24th January 2012.

### Recruitment and description of participants

We used purposive sampling to recruit HCPs working for a range of organisations across London, UK, including care homes and NHS settings such as memory clinics, mental health and commissioning services. We did not set participant inclusion and exclusion criteria such as length of time in their role or organisation as our primary aim was to gather the views of a variety of HCPs working within and providing services to care homes. Interviews took place between September 2012 and October 2013. Potential participants were identified by research staff recruiting residents from care homes in London to a parallel cohort study [18] and through the team’s knowledge of other relevant HCPs. Potential participants were given an information sheet and if they provided their contact details to the research team, they were then contacted by the researcher to arrange an interview. Interviews took place at a location of the participant’s choice, which for the majority of HCPs was at their place of employment. Written informed consent was obtained on the day of the interview. We recruited 14 HCPs of whom eight were based in privately-owned care homes (all with some nursing beds), four were from NHS organisations providing health and social care services to care homes, and two were commissioners for older adult services from the local CCG (*see* Table 1*for participant characteristics*). A total of 13 interviews were conducted; the two participants from the CCG were interviewed together. Although participants had a range of experience and the length of time in their current role was variable (11 months to 14 years), they were all experienced in the health and social care sector. Interviews lasted approximately an hour.

**Table 1:** Participant characteristics

ID	Job title	Gender	Time in current role	NHS/Care home
1.	Care Home Manager	Female	11 months	Care home
2.	Clinical Nurse Manager	Male	7 years	Care home
3.	Care Home Manager	Male	5.5 years	Care home
4.	Heath Care Assistant	Female	Approximately 3.5 years	Care home
5.	Nurse	Female	3 years	Care home
6.	Mental Health Nurse	Male	2 years	Care home
7.	Clinical Manager (Previous Link Nurse)	Female	Unknown	NHS
8.	Admiral Nurse West Sector Lead	Female	14 years	NHS
9.	Health Care Assistant	Female	1.5 years	Care home
10.	Health Care Assistant	Female	9 years	Care home
11.	Approved Mental Health Professional	Female	3 years	NHS
12.	Occupational Therapist (mental health)	Female	7 years	NHS
13.	Head of Service: Integrated Care (Joint Commissioning)^a^	Female	6 months	NHS
	Joint Commissioner Older Adults and Physical Sensory Impairment^a^	Female	3 years	NHS

*Note.* Time in current role data obtained during interview; ^a^Joint interview conducted with participants from CCG

### Realist evaluation

This was a qualitative study in which we used the realist framework of context, mechanisms and outcomes [27] to gain a deeper understanding of palliative care provision for those with advanced dementia residing in care homes. A realist approach enables the exploration of processes currently in place within health and social care contexts and provides data to inform our understandings of what works for whom, in what circumstances and why [29]. It is useful in uncovering underlying context-mechanism-outcome (CMO) configurations which may influence specific causal processes and their outcomes [27]. The interviews were interactive, enabling the interviewer and participant to engage in a creative discussion without the restrictive parameters imposed by tightly structured interview schedules [27]. As our collection of data was purely qualitative in nature, we are aware that the CMO configurations we present include mid-range theories based on our interpretation of the HCPs views on end of life care for residents with advanced dementia. Additionally, although the realist framework is commonly used to evaluate programmes or interventions, the CMO configurations can be applied to disentangle the complexity of providing care to people with advanced dementia and define the contexts and mechanisms which can result in good and poor care outcomes.

### Data collection

A loosely framed topic guide was informed by a literature review, workshops with members of the health and social care sector, carers and people with early dementia, and by information from an ongoing cohort study within the research programme [18].

### Data analysis

All interviews were audio-taped, transcribed verbatim and entered onto a qualitative software programme (Atlas-ti) for coding. To ensure accuracy, the transcripts were checked against the audio recordings. Transcripts were initially read several times to achieve familiarisation with the data prior to analysis and coding using thematic analysis as described by Braun and Clarke [30]. Thus, units of text were broken down into categories to develop appropriate codes that were then grouped together to form themes. We used an iterative process to ensure that each unit of text was assigned to the most relevant code(s) and theme(s) as new categories were identified. For rigour and transparency, we used memos during the coding process. For triangulation, other members of the team reviewed three randomly selected interviews and any differences in the coding framework were resolved through discussion. We then categorised the themes according to context, mechanisms or outcomes, and identified common links between them. Our aim was to explore how the context and mechanisms influenced the outcome of good end of life care. We noted that participants used their own definitions of what constituted good end of life care in dementia. Minor grammatical changes have been made to some quotes for ease of reading.

## Results

We describe the contextual factors followed by an exploration of the factors that participants considered reflected good end of life care; the outcome, with supportive quotes. In the next section, we incorporate our realistic logic of analysis, to present our interpretation of the mechanisms that are triggered by the contexts outlined in the first section. Table 2 summarises the contexts, mechanisms and outcomes separately. In the final section we present the connections and links between contexts, mechanisms and outcomes as mid-range theories.

**Table 2:** Summary of themes and sub-themes for context, mechanisms and outcome

CMO	Theme	Sub-theme
Context	Business driven care homes	• Profit prioritised over care quality
		• Lower staff salaries and lowly skilled care staff
		• Minimal staffing levels
		• Poor staff conditions
		• Increasing turnover of staff
		• Negative image of care homes and low prestige working in care homes
		• Demanding workloads
		• Staff have limited time
	Complex network of health and social care providers	• Multiple agencies to make referrals to and communicate with
		• No option to make direct referrals from care home
		• Long waiting times for some services
		• External HCPs who are proactive and helpful in providing care to people with advanced dementia
	Societal and family attitudes towards care home staff	• Negative perception of care homes
		• Recognition that care home staff work hard
		• Lack confidence in care home staff
	Staff training, experience and reflective processes	• Lack of training/experience in dementia care
		• Post-death reflections
		• Beneficial to prepare staff for end of life care and to provide exposure to end of life care
	Governance and regulation of care homes	• Highly regulated
		• Excessive documentation and scrutiny
	Complexities of providing care in advanced dementia	• Long trajectory and unpredictable prognosis
		• Challenging to manage symptoms due to the communication difficulties
		• Difficult to understanding the relationship with palliative care
		• Palliative care services not equipped to manage behavioural symptoms of dementia
	Advance care planning	• Proactive Advance Care Planning on admission
		• Importance of involving GP and family in these discussions
	Staff personality/characteristics	• Compassion
		• Motivation
		• Initiative
		• Finding the job rewarding
Mechanisms	Level of HCPs confidence	• Confidence/uncertainty about best approach to end of life care
		• Fear of litigation
		• Fear of death (avoidance)/Accepting (comfortable with dying/death)
	Family uncertainty about end of life care	• Confusion/uncertainty regarding end of life care decisions
		• Family avoiding discussions regarding end of life
	Resources for improving end of life care and supporting families	• Admiral nurses
		• Post-death reflections
	CCGs uncertainty about whether dementia specific palliative care is required	• Uncertainty as to whether specific dementia palliative care services are necessary
Outcomes	Psychosocial and spiritual care	• Beyond meeting basic physical needs
		• Person-centred approach
		• Spending time with residents
		• Treated with dignity and respect
		• Being seen by a religious figure e.g. priest
	Supporting and developing relationship with family carers	• Collaboration between family and care home staff
		• CH staff getting to know the family and obtaining trust
		• CH staff helping family and carers prepare for their relative’s death and discussing grief
		• CH staff providing support
	Addressing physical needs	• Symptom management (particularly for pain)
		• Reducing burdensome interventions, hospitalisation and resuscitation
	Continuity of care, integrated care and multidisciplinary care	• Good working relationships across services
		• Regular staff who get to know individual needs of residents

### Context of care

#### Care homes work strictly to a business-driven model within a complex network of providers

The context of care was mostly described in negative terms. Economic factors tended to dominate the discussions about barriers to optimal care. Driven by this business model, care homes were described as profit-driven organisations providing poor staff salaries, employ lowly qualified staff and minimal levels of staffing.

If it hadn’t been that my husband had passed away and left me some money, I couldn’t do this job. This is the job I thrive on, I love. I’d do it 24/7, but if I had to live on the money that I get. I couldn’t do it. And how sad is that? … Six pounds something you get here an hour. (ID: 4)

From my experience – its right across the whole country, you never get enough staff in. It’s only, occasionally you might have three carers to support you, but most of the time, the shortness of the adequate personnel is a crisis. [*Why is that?*] Well, I do believe that one of the reasons is, cost effectiveness… it’s a profit making organisation, that doesn’t make it easy. (ID: 6)

Care homes must rely on a complex network of health and social care providers. Although these services collectively aim to provide a comprehensive package of care, delays to providing care are common and care home staff are required to liaise with multiple agencies to coordinate care without the option for direct referrals.

Yeah, it’s quite good [*the support*], but sometimes it’s the time span. Because if … someone is away, or they’re not on and you’re waiting two or three days, sometimes you need them now, not two weeks’ time, or in an emergency where it’s affecting others. (ID: 10)

So we have a physio [*physiotherapist*] in everyday for the intermediate care unit and occupational therapist for rehab assistance, and then obviously we have physios, private physios, NHS physios, for our other residents… But it’s sort of like frustrating that you can’t refer straight [*to other HCPs*] from the nursing home (ID: 1)

#### Societal and family attitudes towards care home staff

HCPs external to care homes are aware of the negative image of care homes and care home staff portrayed in the media and the following quote highlights that care homes provide poor salaries for demanding workloads.

I’m quite interested in working with homes because they do get a bad press, the staff. And they are low paid and they work very hard, but sometimes I think if they had a bit more time they could be… I’ve seen terrible treatment really, that we’ve reported, of people with dementia and I wonder if it’s caused by low pay, not enough time (ID: 12)

External healthcare professionals and family members appeared to lack confidence in care home staffs’ ability to make end of life care decisions.

Sometimes, you know, they [*family members*] don’t trust… they’re thinking, maybe we are wrong and they want a second opinion… maybe we are wrong [*about not sending the resident to hospital*] and somebody else can say something different from what we’re saying. (ID: 5)

#### Staff training, experience and reflective processes

Care homes employ staff with minimal skills to care for people with complex needs such as those with advanced dementia. Participants commented about the lack of experience and absence of dementia training among those who are recruited to work in care homes.

The majority of the health care assistants, they are just from school or wherever they are from. I mean no training, they just come on the unit. And they are expected to function (ID: 6)

I think it’s [*training*] lacking in every area, in every area. I think it’s lacking in domestic, the domiciliary services that we purchase. I think it’s lacking in the homes that we place people in. (ID: 11)

However, despite the lack of training, several participants reflected on post-death discussions which are common in their work place as a method of improving responses to end of life care.

One of the things that we do is we do ‘after-death analysis’ and ‘significant event’ analysis where when somebody dies we actually go up to the floors and get the nurses and the carers and even the housekeeper or what have you and we sit down and we look at what has happened. Maybe how we could have improved things (ID: 3)

Similarly, with the aim of reducing staff fear of death, some care homes encourage staff to experience caring for someone during the dying process.

We try and get all staff to experience the dying process… that when people are dying, staff go in and sit… It could be shocking; people haven’t seen death before and it could have a really adverse reaction on them. So when they’re exposed to it, the feeling is that, people are going to die in the care home so, feelings that they will be attune to it as something to expect (ID: 3)

#### Governance and regulation of care homes

The care home sector was also seen as being highly scrutinised by regulatory bodies that demand excessive documentation promoting, in response, a fear of litigation (which is discussed below as a negative mechanism triggered by this context).

It’s important that we keep those experienced nurses, because the standards are so high and the accountability in care homes is so tremendous, the reason why we lose people is generally because we can’t, care homes, let me rephrase, care home nurses are not paid as well as NHS (ID: 3)

The paperwork is important, because you’re writing down things about them, so you’re documenting everything about them. But while you’re doing that as well, you’re not with them. So it’s really hard to get a fine line, because it’s not just one person you’re documenting, it’s the whole lot that you’re documenting on. (ID: 9)

#### Complexities of providing care in advanced dementia

Contextual factors referring to the nature of dementia were also discussed. Dementia was described as a complex condition with an unpredictable prognosis, and that there were difficulties assessing and managing symptoms and understanding its relationship to palliative care. Various participants felt that palliative care professionals were generally not prepared for dementia. In particular, palliative care teams found the communication needs and behavioural problems of these residents deeply worrying. They described a ‘gap’ in their provision. Moreover, it was not clear whether this was a simple lack of training and education, or a more profound resistance to dealing with this patient population that was perceived to be difficult and regarded as not within their own professional remit.

Increasingly the patients that are coming through invariably there’s a large proportion that have an element of dementia… they [*palliative care services*] don’t necessarily have the skills to deal with them if they have quite severe dementia. You know, it becomes much more challenging for them, their services are all geared up to what they would define as standard palliative care. If you’ve got someone that has challenging behaviour or has severe dementia then all of those become an awful lot more different. (ID: 13)

#### Advance care planning

Proactive advanced care planning was viewed as minimising unnecessary hospitalisation and burdensome treatments. This was identified as a routine and collaborative process in one care home.

We’d normally do … advanced care plan and do not resuscitate if the GP [*General Practitioner*] and family are happy with that. The GP likes to speak to the family as well so he likes them to be in agreement (ID: 1)

#### Staff personality/characteristics

The compassion, motivation and initiative of care home staff are presented here as contextual factors that impact on the quality of end of life care in both negative and positive ways. Some participants described care home staff as focusing on basic physical care needs, failing to address psychosocial needs. These are the dimensions of staff characteristics that detract from compassionate care:

You know could you really, just tell somebody not to ring their bell, I mean, what happened to kind, caring, compassion, you know, ‘would you like a cup of tea, how about I sit and have a chat with you for a while’ (ID: 1)

This is what I’m saying about carers in general, they won’t motivate their brain, they will do. They will wash, they will clean, they will feed, but not anything in between. (ID: 4)

Staff, nursing staff, identifying things… They are really bad, they know it when you say it to them but you shouldn’t have to be reminding them, they should know. I was going to try and draw up some pathways, I was thinking about it last night, so if this happens, you need to do this, this and this… It will take them a little while. (ID: 1)

However, there were also care home staff who found their job rewarding and wanted to provide good quality care.

It’s rewarding. Like I say, everyone here is different. Every resident is… but it is rewarding because you give them a better quality of life. (ID: 10)

### End of life care outcomes

Our analysis focused on the outcome of good quality end of life care in dementia which was described by participants as: psychosocial and spiritual care, supporting and developing relationship with family carers, addressing physical needs and providing continuous and integrated care by a multidisciplinary team.

#### Psychosocial and spiritual care

Care homes were often viewed as providing a minimal level of service addressing only basic physical needs rather than a quality service that looked after the psychosocial needs of the individual. The following participant highlights this:

I actually think this home does meet their basic needs - they eat well, they look well… they look nice if you go and see them, their rooms are nice and tidy - but the rest of the time, people are just left and I think that that’s a tragedy. That to me is a real tragedy of dementia care, of any sort of care, actually, just to be left (ID: 11)

Participants described the need for care homes to adopt a more person-centred approach.

They are just treated with the dignity and respect that they deserve, and that they’re seen as individuals really… Obviously, the other bits about not being in pain and all that kind of stuff (ID: 11)

#### Supporting and developing relationships with family carers

Similarly, providing support for family carers of people with dementia who are approaching the end of life is a crucial aspect of the care home service. The following quote suggests that some care home staff take the time to get to know families and prepare them for the death of a loved one.

And we know them [*family members*], we get to know them, and we’re around. It’s just like a big family, really. You know dad or mum is going to pass. It’s just a case of when, as long as they’re comfortable. And they know us, the residents know us as well. (ID: 9)

However, there are challenges to providing support to family members and initiating conversations about end of life as this participant suggests that family members do not readily engage with support groups provided by care homes and some actively avoid discussions which may result in making decisions.

We also do that for family members, we offer bereavement groups, although it’s not been regularly attended for, I can’t imagine the reason why that’s been … I think we need to do more work on bereavement. Not just for the staff but also for family members and like I said we have the group but they’re not coming, maybe need to take a look and see why people are not coming. (ID: 3)

Some families just don’t want to discuss it [*death*] … The whole family choices, because they can’t make them. (ID: 10)

#### Addressing physical needs

Although so far we have demonstrated that care home staff fail to provide adequate support to residents due to various contextual factors, there was evidence of good quality end of life care, particularly for managing physical needs. For example, care home staff did manage symptoms such as pain at the end of life well.

Sometimes we phone the doctor up and say, ‘She’s end of life, she’s really in pain,’… An example, there was a lady that was dying downstairs, and I said to [*colleague*], ‘She needs something to calm her down,’ because I don’t have anything to do with that, but I see this lady. She had dementia, and I said… - ‘She needs something to calm her down.’ If she’s going to die, she needs to die peacefully, not all up high. So we phoned the GP, he did write a prescription (ID: 9)

Similarly, some care home staff worked to reduce unnecessary and burdensome interventions, hospitalisation and resuscitation, such as having a clear plan for care for individual residents:

Staff are comfortable when they go on duty they know who is for resuscitation, they know who’s in palliative care, they know who they need to call for the doctor for and they know who they don’t have to call the doctor for or the ambulance for so that staff are very reassured, when you talk to the nurses you’ll find that we don’t have that issue any more. (ID: 3)

#### Continuity of care, integrated care and multidisciplinary care

Another positive outcome is the provision of continuous, integrated care provided by a multidisciplinary team. At a care home level, staff get to know the residents they provide care for:

I’ve got a good bunch of care assistants up here, and they all work very well together, and they get to know the residents. It’s hard when new ones come in, because you’ve got to start all over again, and it takes a while to get to know them. (ID: 9)

Some care home staff had good relationships with external services and provided a continuous service ensuring that residents who required treatment did not wait long.

I wanted to say to you is that we’ve got a very good relationship with the palliative care team (ID: 8)

We have no delay in treatment at [*Care home*], we really run, it’s like a mini-hospital. And one example of that is if a GP sees a resident now, at 9.30 am and prescribes pain medication or antibiotics, they are receiving it by 10.30 am… So there’s absolutely no delay. (ID: 3)

However, conflict and issues did arise between care homes and external service providers due to the negative context of a complex health and social network of services within which a care home must function.

Outside [*external HCPs*] help - this sounds bad, but when you call them, I’ve had a few arguments with them on the phone, because I’m not calling you for my benefit, I’m calling you for this lady’s benefit, and it’s no good giving me an appointment in 3 weeks’ time. I need someone now… (ID: 9)

### Mechanisms triggering care

We identified four main mechanisms which were found to trigger the outcomes in care we have described. These mechanisms were: (i) levels of HCPs confidence, (ii) family uncertainty about end of life care, (iii) resources for improving end of life care and supporting families and (iv) uncertainty within CCG’s as to whether specialist palliative care teams were required to address the complex needs of those with advanced dementia. All four mechanisms are interrelated often reflecting overall uncertainty and a lack of confidence amongst key stakeholders regarding the best approach to care.

#### Levels of HCPs confidence

Despite the use of advanced care plans and DNAR orders, care home staff often lacked the confidence to provide end of life care. At this crucial point care home staff, frightened that they would get into trouble, felt unable to adhere to documented care plans.

Their whole ‘resus’ status has been discussed right so you’ve got it written down.… But even when it has been discussed and agreed that this person should not go to hospital, the staff have still sent that person to hospital and then that person’s ending up dying in hospital and stuff like that you know, or has come to hospital and has been discharged within 24 h back to die you know… It’s just really awful. I think it’s because they don’t feel confident to make a decision to follow. They’re probably going to think ‘oh my god if I don’t do something about it then I’ll get’, I’m convinced, so maybe then the staff need to be much more involved in those decisions, or at least in the whole process of that decision being made (ID: 7)

However, our data also suggested that it was not only care home staff confidence in providing care for people with advanced dementia but also external health care providers who lacked confidence in making decisions.

Junior doctors who are out of hours and we will call in the night for support if needed so if somebody suddenly has spiked a fever and that there was perhaps no medications prescribed for it or somebody got really breathless or in pain, we will contact the out of hours GP, one of the things we found that happened on several occasions was that, hospitalised, despite protestations of the nurse and despite the handover, so that’s happened a few occasions.. [*Why do you think that is?*] A combination of lack of, I’m going to use this word carefully, education, related to this specific group and they’re all fears about repercussions. (ID: 3)

We found that confidence was affected by care home staff attitudes and awareness of issues surrounding death and dying. While some staff were comfortable with the end of life, there were indications that many were not.

Nothing wavers me, you know I was born to do this job so I’m, physically I’m not scared and I will be with someone right, right to the end and after the end, that is me… but I know a nurse and a couple of our other carers and someone has actually passed away, they freak out, they freak out! … You know, they can give them medication or see them, you know that stage just before, but once they’ve actually passed away, whether its mentally, the dead body, whether its mentally that, but they freak out and they can’t come in the room (ID: 4)

#### Family uncertainty about end of life care

A negative mechanism regarding the best approach to care at the end of life was family uncertainty about decisions, such as whether or not a resident should be moved to hospital.

Despite the fact that they [*family*] have signed ‘yes I don’t want mum or dad, or what have you, to go to the hospital, I want dad to finish their life here’ when the time really approaches there is a lot of, some confusion in their mind as to whether they are doing the right [*thing*], or they have decided to do the right decision for that person to say ‘ok yes, let them be in the environment that is familiar to them and to pass on peacefully without any pain, without any suffering or without any discomfort’ (ID: 2)

#### Resources for improving care at the end of life and supporting families

The data highlighted some valuable resources which were used to improve care at the end of life. In this example, a care home used post-death reflection to address spiritual needs.

We never used to do so well in that [*spiritual care*] and that’s one of the things we identified doing after death, significant event analysis. We found that initially a lot of people weren’t seeing the priest or their religion wasn’t being addressed (ID: 3)

Providing end of life support to family carers before the person with dementia has died can help families to prepare for death and for bereavement post death. This is considered a core element of the work of Admiral Nurses, another resource that is available to some care homes when supporting families before death and into bereavement.

We [*Admiral Nurses*] talk about the bereavement issues as a day to day thing; I think it’s really important. I’ve got a family at the moment, I spoke to the son this morning, and he said, ‘Mother, you’re never going to die though are you,’ and he won’t let me talk about it, but I know it’s going to be such a big issue for him. So in my agenda it’s something that needs to be talked about, so that he’s prepared. (ID: 8)

Our main role [*Admiral Nurses*] is to support family carers of people with dementia, and that’s throughout the course of the condition, and also all forms of dementia. (ID: 8)

#### Perspectives of clinical commissioning group’s

Finally, due to the increasing number of people with dementia who will have palliative care needs, there was also some apprehension from the perspective of the commissioners about whether there was a need to differentiate palliative care for people with dementia.

So many people who will need palliative care will have dementia, … So maybe that’s against having a specialist team… And from a commissioning perspective I think there would be a bit of a reluctance to have specific teams targeted around specific individuals. I think increasingly they’re moving away from specifics… I mean dementia is sometimes different because sometimes we do have to ring fence services that are for dementia patients, but in this particular one [*developing specific palliative care teams for dementia*] I think that I would agree with [*colleague*], it would be about how you integrate that within existing teams (ID: 13)

### Context-Mechanism-Outcome (CMO) configurations and realist explanations

In this section we present some of the mid-range theories that we have developed using our realist logic of analysis to understand and link the context, mechanisms and outcomes described above. We bring together the themes presented in Table 2 and present six CMO processes that explain our data. We found a high level of complexity in these data and so we include examples which we consider contribute most to understanding end of life care in advanced dementia in care homes.

#### CMO 1

Whilst governance and regulation of care home practices (*context*) are regarded as a necessity to maintain standards, and quality of care, the context of increasing bureaucracy reduced the time available for staff to spend with residents and limited their capacity (*context*) to provide psychosocial and spiritual care (*outcome*). Participants felt that the paperwork had little to do with improved care standards but rather was motivated by a fear of litigation and being seen as ‘doing things right’ (*mechanism*). This is emphasised by the following quote:

These people up at the top say, ‘Oh, yeah, you have to spend at least ten minutes, half an hour with the residents,’ but then, ‘Stop giving me all this paperwork.’ There’s different paperwork every week. [*Has that increased over the years?*] Yeah, oh, gosh, yeah! Obviously people have got to protect themselves… (ID: 9)

#### CMO 2

Despite a negative care home context of low salaried staff with minimal skills, training and experience (*context*), some care homes encouraged their staff to gain confidence and experience (*mechanism*) in providing care for people at the end of life (*outcome*). The following quote suggests that care home staff were sometimes able to spend time with residents who are dying (*context*) to help them become comfortable with dying and death (*mechanism*) and more able to provide care (*outcome*).

We try and get all staff to experience the dying process… that when people are dying, staff go in and sit… It could be shocking; people haven’t seen death before and it could have a really adverse reaction on them. So when they’re exposed to it, the feeling is that, people are going to die in the care home so, feelings that they will be attune to it as something to expect (ID: 3)

#### CMO 3

Although profit-driven care homes provide low pay for demanding workloads (*context*), care home staff who found their job rewarding were motivated (*context*) and confident in the care they provided (*mechanism*) which resulted in getting to know the family and obtaining family trust (*outcome*). This enabled the possibility of better care (*outcome*).

People that are motivated to care, they’re here because they want to be here, not because they’re getting paid for doing it. And carers aren’t paid that much here… and I think we’ve got a lot of staff that are here because they’re doing it and they enjoy doing it (ID:1)

But most of them [*family carers*], they understand, you know, we have for family which aren’t, that are very tough at the beginning but after seeing how we dealing with them and everything they trusting us and after even they saying thank you for everything, they send us a card and plus invitation to the funeral and everything. (ID: 5)

#### CMO 4

When care homes are proactive in developing advance care plans for people with dementia (*context*), this can trigger confidence in care home staff (*mechanism*) to follow these plans with the knowledge that it is the best approach to end of life care for that person (*outcome*). Thus, adherence to care plans (*context*) would enable care homes to reduce unnecessary and burdensome interventions, hospitalisation and resuscitation (*outcome*). However, despite well-articulated care plans (*context*), situations also arose where staff were not confident (*mechanism*) to enact these and resorted to more conservative approaches to care to try to preserve life (*outcome*). Additionally, our data also revealed that it was not just care home staff who lacked confidence when making decisions about the care of people with advanced dementia (*mechanism*) but even junior doctors sometimes fail to make appropriate decisions (*outcome*) within the context of high accountability (*context*).

#### CMO 5

The nature of dementia and complexity of care issues (*context*) also created some conflicts in what constituted the best approach to care or what was in the best interests of the person with dementia (*outcome*). Different opinions between care home staff and family members (*mechanism*) can create stress for staff in situations where the health of the person they are providing care for is deteriorating. This may lead to sub-optimal care (*outcome*).

No, it’s just, as I say, the different characters. There’s another lady we’ve got that has just stopped eating, all of a sudden. Just won’t eat… her family, and they don’t want her to go into hospital… Yet with me and [*colleague*], it’s like we’re sitting there watching her starving herself to death, and you as carers feel helpless because it could be helped if she went to hospital. Not that we’re saying hospitals are the best place for them all to go, but it’s something that could be helped, but the family do not want her to go to hospital, so for us, it’s a very hard situation… We spoke to a doctor, we spoke to the family, we’ve done what we can do (ID: 10)

#### CMO 6

Although health and social care commissioning teams are uncertain if people with advanced dementia require specialist palliative care (*mechanism*), some care home staff indicated that they had good working relationships with existing specialist palliative care teams (*outcome*). The first of the two quotes highlight CCGs uncertainty regarding specialist palliative care services (*mechanism*) and the latter quote suggests that specialist palliative care for people with advanced dementia may not be necessary (*outcome*) if current palliative care teams are proactive and helpful in providing care to people with advanced dementia (*context*).

We’re in the process of reviewing palliative care pathways at the moment. I have to say it [*palliative care for people with dementia*] did come up as a bit of a gap… And you know, we haven’t got to the point of trying to identify what we should do to manage that… Whether that’s upscaling the teams, or whether it’s a specific team… (ID: 13)

The times that I might bring the palliative care in is to do a general assessment on the individual’s needs, because I mean, they’re fantastic the team out there, and they do jump to it quite quickly. I mean I could phone them today, and I know that by the end of the week they would be there, which is wonderful to have. So they’re very good in things like pain management, so those are the times I might say, please can you just go and see this person at home. And they’re very good at feeding back as well, but then they would discharge (ID: 8)

## Discussion

This study used a realist approach to explore the context and mechanisms for promoting high quality end of life dementia care. With an increasing number of people living and dying with dementia in care homes, understanding what constitutes good end of life care for these residents is of increasing importance. Our findings increase our understanding of the context, mechanism and outcome configurations that are of importance, and are consistent with and provide explanations for our original underlying programme theory. The key elements required for high quality care in advanced dementia described by professionals reflected the holistic needs of residents, including physical, spiritual and emotional needs. This was enhanced by spending time with individuals, getting to know them and their families and thus being able to discuss preferences for future care. Place of care was considered to be important, although there was disagreement regarding what constituted the best care environment.

For those with advanced dementia, the care home context is mainly perceived as having a negative impact on quality of care through low paid staff with minimal skills and training. Contextual factors such as employing low paid, unskilled staff who lack confidence have previously been shown to have a detrimental impact on the provision of palliative care to those with dementia [14, 20, 21, 31–33].

Similar to previous reports, our findings suggest that factors such as the complex health and social care environment within which care homes must function [34] and the unpredictable nature of dementia [5, 6, 35, 36] can create obstacles to managing symptoms and providing end of life care. Research exploring the role of care homes within the NHS suggests that the inconsistencies in the quality of care provided to those with advanced dementia is associated with the variability in collaborative working between teams at a local level [13, 37]. The complex nature of dementia also required highly skilled and experienced staff who were able to identify and manage complex symptoms of residents who may not have the capacity to communicate their needs [38]. Thus, educational sessions for care home staff focusing on the provision of person-centred, palliative care for those with advanced dementia [39] would help facilitate care improvements.

Despite research demonstrating that not-for-profit care homes provide better care over business driven institutions [40, 41] the for-profit nature of care homes may not be readily amenable to change without considerable restructuring and refinancing of the health and social care system in the UK. Balancing the need for increased quality reporting to safeguard against poor care and overly bureaucratic reporting requirements is a continuing challenge for all health and social care sectors. The use of more efficient reporting systems, including applying information technology advances and reducing redundant documentation is worthy of further investigation.

Some staff found the pressure of the job challenging but rewarding and enjoyed working with residents. Although time was rarely available, participants recognised the importance of spending time with residents. Processes for supporting staff to adjust to working in an environment that required caring for people who were dying were identified. However, fear of death amongst staff was also present. Fear of death and limited time to spend with residents are commonly reported issues experienced by those caring for people with dementia in the health and social care sector [38, 42].

While the contextual factors created barriers, mechanisms allowed scope for good quality care. For example, our sample indicated the use of after-death analysis as a mechanism for reflecting on practice to improve outcomes. Similarly, services such as Admiral Nurses were found to provide much needed support for family carers both before the death of a loved one and into bereavement. However, services like Admiral Nurses are not consistently available to everyone and the provision of this service has been found to be unevenly distributed across the UK [25]. Similarly, other mechanisms such as HCPs confidence were found to be variable across services. Some participants suggested that care home staff lack confidence to make appropriate care decisions whilst other participants suggested that external HCPs lack trust in care home staff knowledge of their residents. Uncertainty and lack of confidence appeared to increase burdensome interventions and unnecessary hospitalisation.

As highlighted by our findings, continuous, integrated care provided by a multidisciplinary team of experts is an important part of caring for people with advanced dementia. However, good integrated palliative care is hampered by the isolation of care homes within a complex network of services [34], high staff turnover in these institutions [31] and low levels of staff confidence in discussing and managing end of life care [14, 21, 43]. Those living with dementia are less likely to receive palliative care due to an ambiguous and unpredictable disease progression [35]. HCPs both within care homes and hospitals, may fail to recognise when the patient has entered the dying phase [43]. In addition, recent cohort studies indicate that the end stage may be complex and lengthy and that predictors of death are few and difficult to identify [5, 6, 36].

When reviewing the characteristics of good end of life dementia care described in this paper, it appears that the outcomes would be consistent with the definition of palliative care in dementia as described in the EAPC White paper [26]. It is also consistent with the World Health Organisation’s (WHO) definition of palliative care as “An approach that improves the quality of life of patients and their families facing the problem associated with life-threatening illness, through the prevention and relief of suffering by means of early identification and impeccable assessment and treatment of pain and other problems, physical, psychosocial and spiritual” [44]. What became evident, however, was that the context of dementia created obstacles to the provision of good end of life care and required staff with specific skills and experience in caring for people with advanced dementia. Also, given that the majority of people with dementia die in a care home [1] and the majority of people dying in care homes have dementia [24], it appears that the contextual factors create significant barriers to people with dementia receiving good quality end of life care. Although one participant from a commissioning background queried whether specific end of life care services were required for people with dementia, we argue that rather than developing new services, we need to enhance existing services for people with dementia, particularly care homes, to provide a consistent palliative care approach.

### Strengths and limitations

Our work has a number of strengths including the recruitment of a variety of staff from the health and social care sector including commissioners, the collection of rich data from in-depth interactive interviews and the use of triangulation and consensus meetings between researchers to verify the credibility of the data. Use of the realist framework during the development of the topic schedule ensured a dynamic approach so that data collection was responsive to new knowledge and open to discussions of innovative solutions. Additionally, the use of the realist logic of analyses during the data analysis phase enabled us to conceptualise the findings and to develop a deeper understanding of how the context and mechanisms may hinder or promote the provision of good palliative care in care homes.

Our data were collected from the Greater London area and our findings may not be applicable to those working in other areas of the country or in other countries. However, our results are supported by the work of Goodman [21] and Froggatt [45] in the UK. Despite using purposive sampling, we did recruit a variety of HCPs across different service providers both internal and external to the care home. However, these attitudes may not be applicable to other HCPs such as GPs who have clinical responsibility for those residing in care homes and are the first port of call for care home staff when health concerns arise and those working in other areas of the UK or in other countries. Nor do these data include information collected from family carers. However, in developing the intervention to improve care at the end of life in advanced dementia within our wider programme of research, data from family carers and those working in primary care are included. These results, including views of people with early dementia, will be published separately. Although we did not explore home-based dementia care, we did speak to a range of HCPs including those who work in community-based teams providing care to those both living in their own homes and those residing in care homes. Therefore, some of our findings would also be applicable to those living in their own homes. Finally, the lack of mixed methodology in this study is a limitation when developing CMO configurations [46] and thus the CMO configurations presented in this paper are based on our interpretation of HCPs views of care provided to people with advanced dementia. We plan to report further realist based explanations of factors affecting end of life care in advanced dementia at a later date in which we shall be able to include both quantitative and qualitative data collected through our wider programme of work [18].

### Implications for research, policy and practice

Our work informs two of the research priorities identified by the Alzheimer’s Society Dementia priority setting partnership with the James Lind Alliance [47]. Our findings suggest that research on quality of end of life care for people with dementia needs to focus on developing models that enhance the positive mechanisms and reduce the negative processes such as improving confidence in care home staff and helping care home staff to overcome their fear of death and dying. Although there are many positive mechanisms and approaches functioning at local levels, these are not consistently applied throughout the care home sector across the country. Such improvements could be levered through clearer policy guidance and our paper provides some evidence which could inform UK policy in this area.

Changes at a regulatory level are also required. Regulatory and governance procedures across a care home vary greatly, for example, as clearly demonstrated by our findings, care home staff must complete excessive amounts of documentation reporting every single aspect of care they provide, thus leaving minimal time to spend with residents. In contrast, there are no minimum guidelines for the staff to resident ratio in the UK and Care Quality Commission (CQC) inspectors use resident needs to calculate the number of staff needed for each care home [48] which is a time consuming task. Therefore, care homes in the private sector adopt a standard of providing care at minimal levels. Several changes at an industry level can occur to improve the levels of care currently provided. These include more stringent guidelines, more sanctions when care homes fail to provide good end of life care as well as incentives for care homes that demonstrate high quality care.

For major improvements in the quality of palliative care for people with advanced dementia to occur, changes at a higher systemic level are needed. Care homes function within a complex network of health and social care providers. Contextual changes would require streamlined pathways and integration between care homes and these providers.

Whilst it is not yet possible to influence the progressive nature of dementia, the care home environment can be adapted to assist care home staff in the way they manage the symptoms of residents with advanced dementia. If care home structures supported the provision of more professional development opportunities, provided pay that is reflective of the work performed by care staff and improved staffing levels, care home staff would be better equipped to care for residents approaching the end of life. Of particular importance is increased knowledge and skills in managing the symptoms of dementia.

## Conclusion

Application of the realist framework to our data demonstrated how contextual factors and mechanisms influenced care that is provided at the end of life for people with advanced dementia living in UK care homes. In particular, low pay, unskilled staff who lack confidence and poor coordination of external services, need to addressed. Many staff are dedicated to their roles and deserve to have this recognised by pay levels commensurate with the challenges in their work and the skills they need to address them. More time should be devoted to in-work training if a skilled work force is to be developed for the needs of the future. Staff, managers and commissioners do not always understand the needs for palliative care of people with dementia, nor how to work with relatives. Change requires recognition that these patients are challenging and yet rewarding to care for and that supportive care should be available at a much earlier stage. Perhaps most important of all, is the need for managers and commissioners to prioritise this area of care and use business models that respect the dignity of patients and care home staff.

## Abbreviations

CQC: care quality commission
CCG: clinical commissioning group
CMO: context-mechanism-outcome
DNAR: do not attempt resuscitate
EAPC: European Association for Palliative Care
GP: general practitioner
HCPs: health care professionals
NHS: National Health Service
NIHR: National Institute for Health Research
WHO: World Health Organisation
